# Effects of the glucagon-like peptide-1 receptor agonist liraglutide on systolic function in patients with coronary artery disease and type 2 diabetes: a randomized double-blind placebo-controlled crossover study

**DOI:** 10.1186/s12933-016-0425-2

**Published:** 2016-07-26

**Authors:** Preman Kumarathurai, Christian Anholm, Olav W. Nielsen, Ole P. Kristiansen, Jens Mølvig, Sten Madsbad, Steen B. Haugaard, Ahmad Sajadieh

**Affiliations:** 1 Department of Cardiology, Copenhagen University Hospital of Bispebjerg, Bispebjerg Bakke 23, 2400 Copenhagen, Denmark; 2 Department of Internal Medicine, Copenhagen University Hospital of Amager, Copenhagen, Denmark; 3 Department of Endocrinology, Copenhagen University Hospital of Hvidovre, Copenhagen, Denmark; 4 Clinical Research Center, Copenhagen University Hospital of Hvidovre, Copenhagen, Denmark

**Keywords:** GLP-1, Liraglutide, Coronary artery disease, Diabetes mellitus, Dobutamine stress echocardiography, Left ventricular ejection fraction

## Abstract

**Background:**

Patients with type 2 diabetes (T2D) and coronary artery disease (CAD) have increased risk of cardiac dysfunction. The diabetic heart is characterized by increased fatty acid oxidation and reduced glucose uptake resulting in reduced cardiac efficiency. Glucagon-like peptide-1 (GLP-1) has shown to increase myocardial glucose uptake and to improve myocardial function. We examined the effect of the GLP-1 receptor agonist, liraglutide, on the systolic function of the left ventricle (LV) in patients with T2D and stable CAD.

**Methods:**

In this placebo-controlled crossover study, 41 subjects with T2D and stable CAD were randomized to liraglutide or placebo and underwent dobutamine stress echocardiography (DSE) and exercise tolerance test at beginning and end of each intervention. The primary endpoint was changes in LV ejection fraction. Secondary endpoints were exercise capacity and other measures of systolic function: wall motion score index (WMSI), global longitudinal strain (GLS) and strain rate (GLSR).

**Results:**

Liraglutide, when compared to placebo, did not improve LV ejection fraction at rest (+0.54 %; 95 % CI 2.38–3.45), at low stress (+0.03 %; 95 % CI 3.25–3.32), at peak stress (+1.12 %; 95 % CI 3.45–5.69), or at recovery (+4.06 %; 95 % CI 0.81–8.93). No significant changes in WMSI were observed at any stress levels. GLS and GLSR at rest did not improve. The maximal exercise capacity estimated by metabolic equivalents was not affected by liraglutide.

**Conclusion:**

In conclusion, liraglutide did not improve the systolic function of the left ventricle during DSE or the exercise capacity in patients with T2D and stable CAD.

*Clinical Trial Registration*http://www.clinicaltrials.gov (unique identifier: NCT01595789)

**Electronic supplementary material:**

The online version of this article (doi:10.1186/s12933-016-0425-2) contains supplementary material, which is available to authorized users.

## Background

Type 2 diabetes (T2D) and coronary artery disease (CAD) increases the risk of cardiac dysfunction [1]. Subclinical LV dysfunction is present in patients with T2D and is attributable to factors such as insulin resistance, microvascular disease and cardiac autonomic dysfunction [2]. Despite improved glycemic control in patients with T2D treated with glucose lowering agents, there is little evidence from clinical trials of reduced risk of heart failure, although the EMPA-REG Outcome Study recently has challenged this view [3]. Some glucose lowering agents have been associated with an increased risk of hospitalization for heart failure [4]. Consequently, investigations of safe anti-glycemic treatments that may improve or preserve cardiac function in patients with type T2D and CAD are warranted.

The diabetic heart is characterized by increased fatty acid (FA) oxidation and reduced glucose uptake resulting in a decreased cardiac efficiency as more oxygen is needed to generate ATP. This feature is undesirable in the ischemic setting where oxygen supply is limited [5]. The incretin hormone, glucagon-like peptide-1 (GLP-1), has shown to increase myocardial glucose uptake [6]. This observation has facilitated clinical studies where GLP-1 infusion improved cardiac function in patients with CAD and reduced [7, 8] or preserved systolic function [9]. However, the use of short-term continuous infusion of GLP-1 in these studies limits the clinical application of the findings. Thus, the effect on systolic function using a GLP-1 receptor agonist suitable for long-term treatment was evident. In particularly, this may be important in patients with T2D and CAD as it may affect the long term prognosis.

The long lasting GLP-1 RA liraglutide with a half-life of 13 h has in combination with the biguanide metformin shown to be a safe treatment option in patients with T2D [10]. We hypothesized that treatment with liraglutide added to a backbone therapy of metformin in patients with CAD and T2D would improve the systolic function of the left ventricle during dobutamine stress.

## Methods

This is a randomized, double-blind, placebo-controlled 12 plus 12 week crossover study with a 2 week washout period. The outline of the trial visits, the inclusion criteria and the exclusion criteria have all been described in detail previously [11]. In short, patients with stable CAD, left ventricular ejection fraction (LVEF) >40 % and newly diagnosed T2D within 24 months were identified using patient files from selected hospitals in Copenhagen area and invited consecutively to participate in this study. Patients were enrolled from May 2012 until final data collection in October 2014. The subjects underwent dobutamine stress echocardiography, blood tests, anthropometric measurements, blood pressure measurements, and an exercise tolerance test at the beginning and end of each period (week 0, 12, 14, and 26). The allocation sequence was concealed until all subjects had completed the study and all the echocardiography analyses had been performed.

### Primary and secondary endpoints

The primary endpoint was a change in LVEF assessed by Simpson’s biplane method at rest and during dobutamine stress echocardiography. Secondary endpoints were changes in exercise capacity and changes in other echocardiographic measures of systolic function including: global longitudinal strain (GLS) at rest, global longitudinal strain rate (GLSR) at rest, and wall motion score index (WMSI) at rest and during dobutamine stress echocardiography.

### Study drug and dosage

Subjects had a minimum 2 week washout period for their glucose lowering therapy prior to the first baseline visit. The study drugs liraglutide/placebo subcutaneous injections and metformin tablets were titrated in an identical manner in both periods: 0.6 mg liraglutide/placebo od + 500 mg metformin bid was increased after 14 days to 1.2 mg od + (1000 mg + 500 mg) daily and to 1.8 mg od + 1000 mg bid after 28 days [11]. Efforts were made to give subjects the same dosage of study drug in both periods.

### Ethics and dissemination

This study was approved by the Regional Committee on Biomedical Research Ethics of the Capital Region of Denmark and the Danish Medicines Agency. The study has been carried out in accordance with the ICH-GCP (International Conference on Harmonization-Good Clinical Practice) standards and was monitored by the GCP-unit for eastern Denmark. Written informed consent was obtained from each participant.

### Dobutamine stress echocardiography

The details of echocardiography protocol have been described previously [11]. In short, two-dimensional echocardiography was performed at rest and during dobutamine infusion using a M5S transducer (Vivid E9, GE Vingmed Ultrasound, Horten, Norway) and analyzed off-line (GE EchoPAC V. 112). Dobutamine was administered an incremental regimen to reach target HR for each stress level [12]. LVEF was calculated using the Simpson biplane method [13] from contrast-enhanced images. Baseline images were used as reference for each stress level and for the examinations in the following visits in an attempt to get comparable visualization of the LV. The echocardiography examinations were performed by four investigators (AS, OWN, OK, and PK). Global longitudinal strain (GLS) and strain rate (GLSR) was assessed using 2D speckle tracking and calculated as the average of the peak systolic values for the apical 4-chamber, 2-chamber, and long-axis views. Wall motion score (WMS) was assessed using a 16-segment model of the left ventricle and graded by the following score: 1 = normal or hyperkinetic; 2 = hypokinetic; 3 = akinetic; 4 = dyskinetic or aneurysmal [13]. Wall motion score index (WMSI) was calculated as the sum of the scores divided by the number of segments visualized. An abnormal stress response was defined as stress induced regional wall motion abnormalities (RWMA), either because of increased WMS at peak stress compared to rest (ischemic response) or because of decreased WMS at low stress (biphasic) or peak stress (viable). LV mass index (2D method) and relative wall thickness (RWT) was calculated as recommended [13]. All echocardiography analyses were performed by one observer (PK). Consensus for LVEF was achieved by an average of the LVEF measurements between two observers in any questionable cases. WMS assessment was also reviewed by one senior cardiologist (AS or OWN). If the discrepancy between the observer (PK) and the senior cardiologist involved more than one segment classified as abnormal or more than two points in total WMS score, the images were also assessed by a second senior cardiologist and consensus was obtained.

### Cycle ergometer exercise tolerance test

A standard cycle ergometer exercise tolerance test was performed with a workload appropriate for each subject: a starting work load of 25 W with an increasing work load of 25 or 50 W every 2 min. Subjects were encouraged to exercise until maximal exhaustion. The maximal exercise capacity was expressed as total exercise duration and as estimated metabolic equivalents (METs) [14]: METs = [12 × workload(watt)/weight(kg) + 3.5]/(3.5 ml/kg/min).

### Statistical analyses

Power-calculation has been described previously [11]. A post hoc power calculation analysis was added based on the actual data. The SD for the difference between two values for the measurements in placebo period was used. At rest a SD of 5.0 % was observed, and with n = 30 patients a paired analysis provided 80 % power to detect a minimum detectable difference of 2.7 %. For low stress, peak stress and recovery a SD of 6.5, 7.3 and 9 % was observed and with n = 29, n = 24, and n = 29 this provided 80 % power to detect difference of 3.5, 4.4 and 4.9 %, respectively. A dropout rate of approximately 20 % was estimated. Continuous variables were summarized as the mean ± SD or medians with interquartile ranges, and categorical variables were summarized as percentages. The intention-to-treat (ITT) population was defined as subjects who completed minimum one measurement series in one period. The per-protocol population was defined as subjects who completed both measurement series in both intervention periods. A linear-mixed model with random effects for subjects and fixed effects for period and treatment was used in the analysis of the treatment effect on the primary endpoint in the ITT-population. The paired t test was used to compare the treatment effects of liraglutide and placebo for the per-protocol population. For non-normally distributed data Wilcoxon signed-rank test was used. Subgroup analyses were performed according to baseline DSE response. A two-sided p value of less than 0.05 was considered statistically significant. Reproducibility for LVEF was assessed in four randomly selected patients with four levels of stress (baseline, low stress, peak stress and recovery), totaling 16 measurements per observer. Interobserver variability for three observers (PK, OWN, AS) and intraobserver variability for one observer (PK) were calculated by obtaining variance estimates using two-way and one-way analysis of variance (ANOVA) and were expressed as a coefficient of variation (CoV) and a coefficient of repeatability (COR) [15, 16]. All statistical analyses were performed with Stata 13.1 (StataCorp, TX, USA).

## Results

**Fig. 1:**
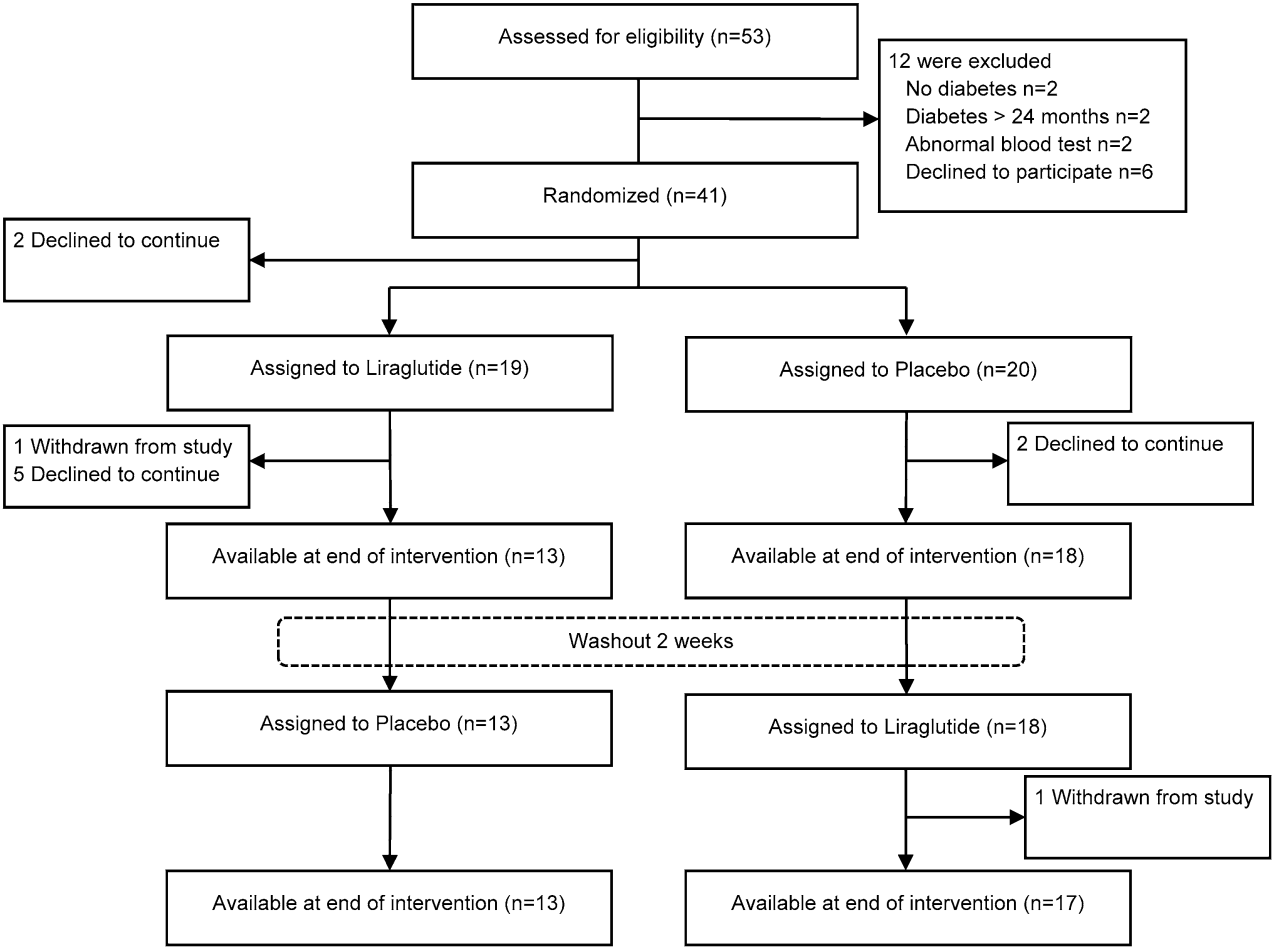
Screening, enrollment, and follow-up of the study population

In total, 41 patients were randomized and assigned to an intervention. Two subjects declined to participate after randomization, leaving 39 subjects available for baseline visit with 19 subjects receiving liraglutide first and 20 subjects receiving placebo first. Subsequently, 9 subjects discontinued the study due to serious adverse events (n = 2), intolerance to medication (n = 3), and other reasons (n = 4). Thus, 30 subjects were available for per protocol analysis (Fig. 1).

Table 1 shows the baseline characteristics for the study population. Subjects had normal LVEF and normal LV geometry. Prior to enrolment in the study, 24 patients were not receiving diabetes medication and were being treated with diet and life style therapy. Fifteen patients were on metformin, and one patient was also receiving sulfonylurea therapy. All subjects had CAD defined by one or more of the following conditions: previous myocardial infarction (MI) (n = 23), previous CABG (n = 13), previous PCI (n = 25) or stenosis >50 % of a major coronary artery (n = 2) (Table 1).

**Table 1:** Baseline characteristics of the study population

Characteristics	Total (n = 39)
Clinical characteristics	
Age, years	61.8 (7.6)
Male sex, n (%)	31 (79)
Weight, kg	96.9 (17.1)
BMI, kg/m^2^	31.6 (4.8)
Waist, cm	110.4 (11.2)
Systolic blood pressure, mmHg	139.3 (19.4)
Diastolic blood pressure, mmHg	80.2 (10.1)
Heart rate, bpm	71.7 (12.1)
Risk factors	
Smoker, n (%)	14 (36)
Hypertension, n (%)	29 (74)
Coronary artery disease	
Previous MI, n (%)	23 (59)
Previous CABG, n (%)	13 (33)
Previous PCI, n (%)	25 (64)
Coronary stenosis, medical therapy only, n (%)	2 (5)
Biochemistry	
Fasting blood glucose, mmol/L	6.5 (1.4)
HbA1C, %	6.4 (0.5)
LDL-cholesterol, mmol/L	2.3 (0.7)
eGFR, ml/min	80.5 (11)
HOMA IR, median (IQR)	4.02 (2.96, 7.49)
f-Insulin, median (IQR), pmol/L	93 (64, 155)
Medication	
Beta blockers, n (%)	24 (62)
Calcium antagonists, n (%)	21 (54)
ACE-I, ARB, n (%)	26 (67)
Statins, n (%)	37 (95)
Ivabradine, n (%)	1 (3)
Diuretics, n (%)	11 (28)
Nitrate, n (%)	11 (28)
Aspirin, n (%)	37 (95)
Pre-study diabetes medication	
Biguanide (metformin), n (%)	15 (38)
Sulfonylurea, n (%)	1 (3)
Diet and lifestyle therapy only, n (%)	24 (62)
Echocardiographic measures	
LVEF, %	58.9 (7.6)
LVmass index, g/m^2^	81.9 (21.4)
RWT, cm	0.33 (0.09)

Data are expressed as the mean (SD), n (%) or median (quartiles 1–3)

*ACE-I* angiotensin converting enzyme inhibitor, *ARB* angiotensin receptor blocker, *BMI* body mass index, *bpm* beats per minute, *CABG* coronary artery bypass grafting, *eGFR* estimated glomerular filtration rate, *HbA1C* glycated hemoglobin, *HOMA-IR* homeostasis model analysis of insulin resistance, *MI* myocardial infarction, *LDL* low-density lipoprotein, *LVEF* left ventricular ejection fraction, *RWT* relative wall thickness, *PCI* percutaneous coronary intervention

### Effect of liraglutide on systolic function

Table 2 shows the treatment effect of liraglutide and placebo on systolic function for patients with a complete measurement series at each stress level. Changes in LVEF during liraglutide period were not significantly different from changes during placebo period at any stress level. No improvement in WMSI at any stress level was observed. No significant changes were evident for GLS and GLSR. ITT analysis revealed a near-significant change in LVEF at recovery (coefficient 4.10, 95 % CI 0.01–8.20). No significant changes were observed in the other stress levels (Additional file 1: Table S1).

**Table 2:** Effect of liraglutide versus placebo on systolic function at each stress level

	Before liraglutide	After liraglutide	Before placebo	After placebo	N	Treatment effect		Difference (95 % CI)	p value
	Baseline	12 weeks	Baseline	12 weeks		Liraglutide	Placebo		
LVEF, %									
Rest	59.46 (7.44)	60.13 (9.07)	59.27 (7.92)	59.41 (7.92)	30	0.67 (6.30)	0.13 (4.95)	0.54 (−2.38 to 3.45)	0.710
Low stress	70.73 (9.58)	71.03 (10.7)	71.31 (9.51)	71.44 (8.43)	29	0.28 (6.27)	0.25 (6.56)	0.03 (−3.25 to 3.32)	0.984
Peak stress	75.72 (9.52)	76.92 (9.14)	76.26 (8.72)	76.02 (8.12)	24	0.87 (6.91)	−0.25 (7.26)	1.12 (−3.45 to 5.69)	0.618
Recovery	60.65 (10.38)	64.91 (10.44)	62.52 (10.20)	62.84 (9.88)	29	4.25 (7.13)	0.20 (8.97)	4.06 (−0.81 to 8.93)	0.099
WMSI									
Rest	1.088 (0.155)	1.084 (0.163)	1.088 (0.144)	1.075 (0.140)	30	−0.003 (0.065)	−0.013 (0.055)	0.009 (−0.018 to 0.037)	0.492
Low stress	1.078 (0.202)	1.077 (0.171)	1.059 (0.143)	1.048 (0.128)	29	0.004 (0.097)	−0.011 (0.056)	0.007 (−0.035 to 0.049)	0.725
Peak stress	1.079 (0.235)	1.052 (0.160)	1.058 (0.149)	1.060 (0.213)	24	−0.03 (0.10)	0.01 (0.08)	−0.038 (−0.112 to 0.035)	0.292
Recovery	1.090 (0.198)	1.096 (0.212)	1.081 (0.157)	1.090 (0.192)	29	0.007 (0.07)	0.006 (0.11)	0.001 (−0.045 to 0.047)	0.963
GLS									
Rest	16.26 (2.60)	15.53 (2.72)	16.44 (3.07)	16.34 (2.90)	30	−0.73 (1.87)	−0.10 (1.87)	−0.63 (−0.42 to 1.67)	0.231
GLSR, s^−1^									
Rest	0.90 (0.18)	0.91 (0.18)	0.86 (0.17)	0.88 (0.2)18	30	0.01 (0.15)	0.02 (0.14)	−0.01 (−0.05 to 0.08)	0.713

Data are expressed as the mean (SD)

*N* is the number of subjects for each treatment phase with valid measurements in each stress level, *GLS* global longitudinal strain, *GLSR* global longitudinal strain rate, *WMSI* wall motion score index, *LVEF* left ventricular ejection fraction

Liraglutide did not affect any significant changes in LV end-diastolic and end-systolic volume (Additional file 1: Table S2). Nine subjects in the per-protocol population had abnormal stress response at baseline and sub-group analyses did not reveal any significant changes in LVEF at any stress level (Additional file 1: Table S3).

### Effect of liraglutide on exercise test performance

Exercise tolerance test results were available for 21 subjects for all 4 visits. No significant differences in the maximal achieved METS was observed between the liraglutide and placebo treatment period (METS −0.13 ± 0.6 vs. −0.42 ± 0.87; 95 % CI −0.22 to 0.80; p = 0.244). The total exercise time was slightly reduced at the end of both treatment periods, but with no difference in the treatment effect (−25 vs. −24 s; p = 0.980, respectively).

### Effect of liraglutide on metabolic and hemodynamic parameters

Liraglutide induced a significant weight loss (−3.2 kg; 95 % CI −4.8 to −1.6; p < 0.001), reduction in waist ratio (−2.2 cm; 95 % CI −4.1 to −0.3; p = 0.026), and reduction in HbA1C (−0.4 %; 95 % CI −0.6 to −0.2; p < 0.001) in comparison to placebo. No significant changes in LDL-cholesterol, triglyceride, HOMA IR, insulin or fasting blood glucose were observed (Table 3).

**Table 3:** Effect of liraglutide versus placebo on anthropometric and biochemical variables

	Treatment effect		Difference	95 % CI	p value
	Liraglutide	Placebo			
Weight, kg	−4.17 (3.49)	−0.98 (2.62)	−3.18 (4.31)	−4.79 to −1.57	<0.001
Waist, cm	−2.80 (4.11)	−0.57 (2.52)	−2.22 (4.89)	−4.16 to −0.29	0.026
BMI, kg/m^2^	−1.35 (1.10)	−0.31 (0.85)	−1.04 (1.34)	−1.54 to −0.54	<0.001
Systolic blood pressure, mmHg	−8.10 (17.27)	−3.17 (16.07)	−4.93 (23.68)	−13.78 to 3.91	0.263
Diastolic blood pressure, mmHg	−3.13 (12.11)	−3.83 (8.75)	0.70 (17.06)	−5.67 to 7.07	0.826
HbA1C, %	−0.42 (0.34)	−0.04 (0.43)	−0.37 (0.54)	−0.57 to −0.17	<0.001
LDL-cholesterol, mmol/L	−0.25 (0.72)	−0.17 (0.63)	−0.08 (0.96)	−0.47 to 0.30	0.657
HOMA IR, pmol/L	−1.35 (3.18)	−0.57 (2.41)	−0.78 (3.24)	−2.01 to 0.45	0.336
Fasting plasma insulin, pmol/L	−11.73 (57.54)	−1.69 (43.79)	−10.04 (68.27)	−36.01 to 15.93	0.469
Fasting blood glucose, mmol/L	−0.99 (1.11)	−0.62 (0.96)	−0.36 (1.06)	−0.76 to 0.03	0.125

Data are expressed as the mean (SD)

*BMI* body mass index, *HbA1C* glycated hemoglobin, *LDL* low-density lipoprotein, *HOMA IR* homeostasis model analysis of insulin resistance

Systolic and diastolic blood pressure measurements at study visits or during dobutamine stress did not change significantly; however, a significant increase in the resting heart rate was observed (6.2 beats per minute (bpm); 95 % CI 0.8–11.5; p = 0.001). Heart rate was also increased after liraglutide treatment at low stress (10.3 bpm; 95 % CI 0.2–20.4; p = 0.046) and peak stress levels (5.3 bpm; 95 % CI 1.2–9.5; p = 0.014) (Additional file 1: Table S4).

### Reproducibility

For LVEF measurement the interobserver and intraobserver variability were, SD 3.4 and 2.2 %; CoV 4.4 and 2.9 %; COR 6.6 and 6.1 %, respectively.

### Safety and compliance

The frequency of adverse events was higher in the liraglutide treatment period and was predominantly due to gastrointestinal side effects: nausea (20 %), anorexia (13 %), and gastroesophageal reflux disease (9 %) (Table 4; Additional file 1: Table S5). A total of 9 serious adverse events (SAE) were observed in the study period (Additional file 1: Table S6). Compliance to liraglutide and placebo was high, and there was no difference between treatment periods (94.4 and 93.6 %; p = 0.66). No difference in compliance to metformin was observed (94.4 and 90.9 %; p = 0.07).

**Table 4:** Adverse events in each period by event category

Event category	Liraglutide, n (%)	Placebo, n (%)	Washout, n (%)
Abnormal blood test	4 (4.4)	1 (2.6)	1 (7.7)
Cardiac	8 (8.8)	6 (15.8)	4 (30.8)
Gastrointestinal	56 (61.5)	15 (39.5)	2 (15.4)
Infection	4 (4.4)	5 (13.2)	3 (23.1)
Muscle	0 (0)	1 (2.6)	1 (7.7)
Neurological	11 (12.1)	6 (15.8)	2 (15.4)
Renal, urine	1 (1.1)	1 (2.6)	0 (0)
Skin	3 (3.3)	1 (2.6)	0 (0)
Vascular	2 (2.2)	2 (5.3)	0 (0)
Other	2 (2.2)	0 (0)	0 (0)
Total adverse events	91	38	13

Data are expressed as n (%)

## Discussion

In this double-blind, placebo controlled study we showed that 12 weeks of liraglutide treatment did not improve LVEF during dobutamine stress in patients with stable CAD, preserved LVEF and newly diagnosed T2D. Furthermore, no significant changes in WMSI or exercise capacity were observed. GS and GSR parameters at rest did not improve either.

Early studies of GLP-1 infusion were performed in patients with heart failure and showed an improvement in LVEF after both short term and long term GLP-1 infusion [7, 17]. However, these findings could not be confirmed in randomized placebo-controlled studies. Two days of GLP-1 infusion did not improve LVEF in non-diabetic patients with chronic compensated heart failure [18]. Recently, a randomized study showed no effect of 12 weeks of treatment with the GLP-1 RA albiglutide on cardiac function in patients with heart failure [19]. Although the subjects in the two latter studies did not have T2D and had reduced LVEF, the findings are consistent with results in the present study.

In patients with preserved LVEF, GLP-1 infusion during coronary artery bypass grafting did not improve postoperative LVEF in a cohort of diabetic and non-diabetic patients [20]. However, 1 week of subcutaneous liraglutide treatment after MI in subjects with and without diabetes showed an improvement in LVEF at 3 months follow-up [21, 22]. In contrast to our study, these studies were performed in a setting of acute MI and thus under stress-induced hyperglycemia and inflammatory response, that might be subject to a particular beneficial effect of GLP-1 RA treatment [23].

Read et al. [9] performed DSE in 14 subjects with preserved left ventricular function awaiting coronary revascularization and showed a significant improvement in LVEF at peak stress during GLP-1 infusion and an attenuation of post-ischemic dysfunction. Accordingly, we would have expected some GLP-1 RA mediated improvement in the systolic parameters in our study cohort during DSE. However, in the study by Read et al. the beneficial effect of GLP-1 was predominantly in ischemic segments. Our subgroup analysis did not show any distinct effect on LVEF in subjects with abnormal stress response. Notably, the group accounted for just nine subjects. Our study probably reflects a clinical relevant situation where most patients with diabetes and CAD have undergone revascularization leading to a low frequency of abnormal stress response. Although microcirculation may still be compromised in these patients, we did not observe any positive effects on LV systolic function by liraglutide treatment. Furthermore, dobutamin stress would increase the myocardial oxygen demand through its inotropic and chronotropic effects mediated by myocardial beta-receptors [24]. The combination of the increased oxygen demand and the less energy efficient FA oxidation, which is increased in the diabetic heart, is expected to reduce the cardiac efficiency [5]. Thus, a shift towards increased glucose-oxidation mediated by liraglutide would potentially improve LV performance. However, this was not evident in our study.

Interestingly, the largest improvement in LVEF in the present study was observed during recovery period after peak stress. In addition to increased myocardial glucose uptake, GLP-1 may activate pro-survival intracellular signaling pathways that may have beneficial effects in particularly in post-ischemic myocardial function [25]. Furthermore, additional pathways such as cyclic adenosine monophosphate mediated activation of protein kinase A [26] may also be involved in the infarct-size reducing effects of GLP–1 RAs as demonstrated in studies using exenatide infusion [27, 28].

GLS assessed by 2D speckle-tracking allows for an angle-independent assessment of systolic function and has shown to be a very sensitive marker for early detection of myocardial disease [29]. Despite preserved LVEF our study population had a reduced GLS compared to a previous suggested reference value of −19.7 % thus reflecting a subclinical systolic dysfunction that characterizes the T2D and CAD population [30, 31].

We observed a significant reduction in HbA1C and a net body weight loss that was comparable to what has been reported in previous studies on liraglutide [32]. It could be contemplated that the newly diagnosed T2D in our study population may have limited the potential effect of GLP-1 agonism on the cardiac function. However, previous studies have shown improvement in cardiac function in cohorts consisting of both T2D and non-T2D patients [7, 9, 21]. Furthermore, myocardial insulin resistance have shown to be an inherent feature of both CAD and T2D [33], and thus providing a treatment target for liraglutide.

GLP-1 infusion has shown to improve remodeling in pre-clinical studies [34]. This was also demonstrated in a retrospective study assessing remodeling by cardiac MRI in patients treated with liraglutide [35]. However, consistent with our study, no improvement in LVEF was observed after 6 months.

Previous reporting of elevated heart rate associated with liraglutide treatment was also observed in our study [36]. In particularly, this has been of concern due to the association between elevated HR and increased risk of LV dysfunction and heart failure [37]. Despite a significant increased resting HR during 12 weeks of liraglutide treatment, we did not find any significant worsening of LV function at rest or during DSE. Interestingly, HR was also increased during low-stress and peak-stress stages of DSE after liraglutide. The chronotropic effect of GLP-1 RAs is believed to be mediated via GLP-1 receptors located to the sinoatrial node [38]. However, studies have suggested that GLP-1 may exhibit an inhibitory effect on sympathovagal balance as well [39].

Exercise capacity is an independent predictor of all-cause mortality and CV-mortality [40]. An improvement in exercise capacity assessed by a 6 min walk test was found after 5 weeks of GLP-1 infusion in patients with heart failure [8], but could not be confirmed in a randomized study where cardiopulmonary exercise testing was performed after 48 h GLP-1 infusion [18] or after 12 weeks of albiglutide treatment [19]. Despite the significant weight loss during liraglutide treatment in our study, we did not observe any improvement in exercise capacity assessed by METS or the total exercise time.

### Safety and adverse events

In general the study drugs were well-tolerated as only three subjects discontinued the study due to intolerance to medication. Two patients experienced MI during the study and were subsequently withdrawn from the study. Both of these events were deemed by the investigators to be unrelated to treatment. One patient experienced an allergic reaction during the DSE at baseline visit. This patient subsequently underwent ergometer stress echocardiography without contrast during all four visits.

### Strengths and limitations

Although comparable DSE scans were aimed for at all four levels of stress and between visits by using reference images from the baseline visit, we cannot exclude that some differences in the scanning positions may have contributed to the overall variation. However, the use of contrast-enhanced 2D echo has shown to increase the accuracy and reproducibility echocardiographic examinations [41]. The homogeneity of our study populations limits the application of our results to other patient groups such as patients with heart failure or patients with longer duration of diabetes and poor glycemic control.

### Perspectives

The presence of subclinical LV dysfunction and increased risk of heart failure in subjects with T2D and CAD necessities exploration of glucose lowering medication that may improve and prevent further deterioration of cardiac function. In this context, an understanding of how GLP-1 RAs affect the myocardial function during conditions of myocardial stress is of great importance. Any improvement in systolic function during stress may prevent the long-term deterioration of LV performance and progression to heart failure. Although, no significant improvement in systolic function was observed in our study, we did not observe any deterioration in cardiac function. Thus, liraglutide may be a safe treatment option in patients with cardiac risk factors and preserved LVEF. The recently published LEADER trial showed improved effect on CV mortality after liraglutide treatment [42]. Notably, no increased risk of hospitalization for heart failure was observed. We believe that our study adds to the further understanding of the results from the various long-term GLP-1 RA trials.

## Conclusions

In patient with preserved LVEF, T2D, and stable CAD, the addition of liraglutide to the backbone therapy of metformin did not improve the systolic function of the LV or the exercise capacity.

## Additional file

10.1186/s12933-016-0425-2 Additional tables.

## Abbreviations

ANOVA: analysis of variance
BMI: body mass index
CABG: coronary artery bypass grafting
CAD: coronary artery disease
CoV: coefficient of variation
COR: coefficient of repeatability
CV: cardiovascular
DSE: dobutamin stress echocardiography
eGFR: estimated glomerular filtration rate
GLP-1: glucagon-like peptide-1
GLS: global longitudinal strain
GLSR: global longitudinal strain rate
HbA1C: glycated hemoglobin
HR: heart rate
ICH-GCP: International Conference on Harmonization-Good Clinical Practice
ITT: intention-to-treat
LDL: low-density lipoprotein
LVEF: left ventricular ejection fraction
METs: estimated metabolic equivalents
MI: myocardial infarction
PCI: percutaneous coronary intervention
RWT: relative wall thickness
T2D: type 2 diabetes
WMSI: wall motion score index
