# Supplementary material for: Effects of the glucagon-like peptide-1 receptor agonist liraglutide on systolic function in patients with coronary artery disease and type 2 diabetes: a randomized double-blind placebo-controlled crossover study
Source: Cardiovasc Diabetol. 2016 Jul 26;15:105. doi: 10.1186/s12933-016-0425-2 (PMC4960858; doi:10.1186/s12933-016-0425-2)
Supplement: Supplementary file 1 — 10.1186/s12933-016-0425-2 Additional tables. [file 12933_2016_425_MOESM1_ESM.docx]

**Additional Tables**

**Table S1. Intent-to-treat analysis for the primary endpoint of changes in left ventricular ejection fraction**

|  |  |  |  |
| --- | --- | --- | --- |
|  | **n** | **Difference vs. placebo**  **Coefficient (95% CI)** | **p-value** |
| LVEF, % |  |  |  |
| Rest | 31 | 0.421 (-2.43 to 3.27) | 0.772 |
| Low Stress | 31 | 0.132 (-3.02 to 3.29) | 0.935 |
| Peak Stress | 29 | 1.076 (-2.72 to 4.87) | 0.578 |
| Recovery | 31 | 4.10 (-0.01 to 8.20) | 0.050 |
|  |  |  |  |

Data are expressed as coefficient (95% CI) from linear mixed model.

**Table S2. Effect of liraglutide versus placebo on left ventricular volume**

|  | **Before**  **Liraglutide** | **After**  **Liraglutide** | **Before**  **Placebo** | **After**  **Placebo** |  | **Treatment effect** | | **Difference**  **(95% CI)** |  |
| --- | --- | --- | --- | --- | --- | --- | --- | --- | --- |
|  | **Baseline** | **12 weeks** | **Baseline** | **12 weeks** | **N** | **Liraglutide** | **Placebo** |  | **p-value** |
| EDV, ml |  |  |  |  |  |  |  |  |  |
| Rest | 100.75 (35.12) | 96.90 (39.64) | 101.54 (37.31) | 97.24 (29.36) | 30 | -3.85 (20.75) | -4.29 (24.64) | 0.44 (-11.89 to 12.77) | 0.942 |
| Low Stress | 96.42 (29.71) | 88.92 (33.03) | 94.38 (29.16) | 92.22 (29.75) | 29 | -7.65 (15.71) | -2.23 (18.10) | -5.42 (-14.94 to 4.10) | 0.254 |
| Peak Stress | 82.38 (27.09) | 80.19 (25.37) | 84.06 (27.28) | 78.43 (25.20) | 24 | -0.64 (14.01) | -5.35 (22.25) | 4.71 (-5.13 to 14.55) | 0.333 |
| Recovery | 93.81 (36.12 | 93.95 (30.62) | 94.24 (28.40) | 96.10 (30.46) | 29 | -0.54 (17.19) | 1.64 (18.97) | -2.18 (-12.83 to 8.46) | 0.678 |
| ESV, ml |  |  |  |  |  |  |  |  |  |
| Rest | 42.54 (23.09) | 40.47 (25.34) | 42.25 (21.38) | 40.27 (18.35) | 30 | -2.07 (12.05) | -1.98 (13.38) | -0.09 (-7.00 to 6.81) | 0.978 |
| Low Stress | 30.03 (19.77) | 27.35 (20.12) | 28.64 (17.36) | 27.61 (16.90) | 29 | -2.77 (7.80) | -1.16 (10.24) | -1.61 (-7.09 to 3.87) | 0.552 |
| Peak Stress | 21.02 (14.59) | 19.96 (15.07) | 21.41 (15.40) | 19.80 (13.30) | 24 | -0.52 (6.49) | -1.52 (9.65) | 1.00 (-4.68 | 0.719 |
| Recovery | 38.84 (25.47) | 35.39 (22.94) | 36.54 (17.57) | 37.12 (20.56) | 29 | -3.66 (9.34) | 0.55 (13.64) | -4.21 (-11.47 to 3.04) | 0.244 |

Data are expressed as the mean (SD).N is the number of subjects for each treatment phase with valid measurements in each stress level.
EDV, end-diastolic volume; ESV, end-systolic volume

**Table S3** Changes in left ventricular ejection fraction by in patients with abnormal dobutamine stress response

|  | Treatment effect | | Difference | p-value | N |
| --- | --- | --- | --- | --- | --- |
|  | Liraglutide | Placebo |  |  |  |
|  |  |  |  |  |  |
| LVEF, % |  |  |  |  |  |
|  |  |  |  |  |  |
| Rest | -0.01 (5.76) | 0.65 (5.43) | -0.66 (-7.01 to 5.70) | 0.817 | 9 |
| Low Stress | -1.87 (6.40) | 0.63 (4.83) | -2.50 (-7.45 to 2.44) | 0.277 | 9 |
| Peak Stress | 2.66 (6.06) | -1.24 (6.39) | 3.90 (-3.86 to 11.65) | 0.273 | 8 |
| Recovery | 3.43 (7.13) | 0.73 (10.19) | 2.70 (-7.53 to 12.93) | 0.560 | 9 |
|  |  |  |  |  |  |

LVEF, left ventricular ejection fraction

**Table S4. Changes in dobutamine stress hemodynamic**

|  | **Treatment effect** | | **Difference** | **95% CI** | **p-value** |
| --- | --- | --- | --- | --- | --- |
|  | **Liraglutide** | **Placebo** |  |  |  |
|  |  |  |  |  |  |
| Systolic blood pressure, mmHg |  |  |  |  |  |
| Rest | -9.0 (17.9) | -6.6 (24.5) | -2.4 (29.0) | -13.4 to 8.6 | 0.657 |
| Low Stress | -7.3 (18.5) | -7.5 (19.6) | 0.2 (29.8) | -12.1 to 12.5 | 0.974 |
| Peak Stress | 6.0 (21.9) | -0.9 (23.3) | 6.8 (37.7) | -9.1 to 22.8 | 0.384 |
| Recovery | -1.2 (26.7) | -4.2 (21.9) | 3.0 (37.7) | -11.6 to 17.6 | 0.680 |
| Diastolic blood pressure, mmHg |  |  |  |  |  |
| Rest | -2.0 (9.5) | -2.9 (11.1) | 0.9 (15.9) | -5.2 to 7.0 | 0.764 |
| Low Stress | -3.8 (12.4) | -6.4 (11.1) | 2.6 (15.3) | -3.8 to 8.9 | 0.412 |
| Peak Stress | -5.0 (19.5) | -0.9 (10.3) | -4.1 (22.5) | -13.6 to 5.4 | 0.378 |
| Recovery | 1.5 (11.7) | -2.1 (11.1) | 3.6 (17.2) | -3.0 to 10.3 | 0.276 |
| Heart rate, bpm |  |  |  |  |  |
| Rest | 6.6 (10.6) | 0.5 (8.1) | 6.2 (14.4) | 0.8 to 11.5 | 0.001 |
| Low Stress | 10.1 (16.9) | -0.2 (14.9) | 10.3 (26.6) | 0.2 to 20.4 | 0.046 |
| Peak Stress | 3.3 (9.5) | -2.0 (9.4) | 5.3 (9.8) | 1.2 to 9.5 | 0.014 |
| Recovery | 4.5 (14.3) | -1.2 (11) | 5.7 (18.3) | -1.3 to 12.6 | 0.106 |
|  |  |  |  |  |  |

Data are expressed as the mean (SD).
bpm, beats per minute

**Table S5. List of total adverse events in study.**

| **Group** | **Event** | **Liraglutide period** | **Placebo period** | **Washout period** |
| --- | --- | --- | --- | --- |
| Abnormal blood test | Amylase increased | 1 (1.1) | 0 (0) | 0 (0) |
| Abnormal blood test | Anemia | 1 (1.1) | 0 (0) | 0 (0) |
| Abnormal blood test | Creatinine Increased | 1 (1.1) | 0 (0) | 1 (7.7) |
| Abnormal blood test | PSA Elevated | 1 (1.1) | 1 (2.6) | 0 (0) |
| Cardiac | Atrial Fibrillation | 0 (0) | 1 (2.6) | 1 (7.7) |
| Cardiac | Non Cardiac Chest Pain | 0 (0) | 0 (0) | 2 (15.4) |
| Cardiac | Chest Pain Cardiac | 2 (2.2) | 3 (7.9) | 1 (7.7) |
| Cardiac | Dyspnea | 1 (1.1) | 1 (2.6) | 0 (0) |
| Cardiac | Myocardial Infarction | 2 (2.2) | 0 (0) | 0 (0) |
| Cardiac | Palpitations | 1 (1.1) | 1 (2.6) | 0 (0) |
| Cardiac | Sinus Tachycardia | 2 (2.2) | 0 (0) | 0 (0) |
| Gastrointestinal | Anorexia | 12 (13.2) | 1 (2.6) | 0 (0) |
| Gastrointestinal | Bloating | 1 (1.1) | 0 (0) | 0 (0) |
| Gastrointestinal | Constipation | 5 (5.5) | 2 (5.3) | 0 (0) |
| Gastrointestinal | Dehydration | 0 (0) | 1 (2.6) | 0 (0) |
| Gastrointestinal | Diarrhea | 7 (7.7) | 4 (10.5) | 1 (7.7) |
| Gastrointestinal | Gastroesophageal Reflux Disease | 8 (8.8) | 1 (2.6) | 0 (0) |
| Gastrointestinal | Gastroenteritis | 0 (0) | 1 (2.6) | 1 (7.7) |
| Gastrointestinal | Nausea | 18 (19.8) | 3 (7.9) | 0 (0) |
| Gastrointestinal | Stomach Pain | 1 (1.1) | 2 (5.3) | 0 (0) |
| Gastrointestinal | Vomiting | 4 (4.4) | 0 (0) | 0 (0) |
| Infection | Chills | 1 (1.1) | 0 (0) | 0 (0) |
| Infection | Infection eye | 0 (0) | 1 (2.6) | 0 (0) |
| Infection | Infections Other Cold | 1 (1.1) | 3 (7.9) | 1 (7.7) |
| Infection | Sinusitis | 2 (2.2) | 1 (2.6) | 0 (0) |
| Infection | Skin Infection | 0 (0) | 0 (0) | 2 (15.4) |
| Muscle | Myalgia | 0 (0) | 1 (2.6) | 1 (7.7) |
| Neurological | Dizziness | 5 (5.5) | 3 (7.9) | 2 (15.4) |
| Neurological | Erectile Dysfunction | 0 (0) | 1 (2.6) | 0 (0) |
| Neurological | Headache | 2 (2.2) | 0 (0) | 0 (0) |
| Neurological | Insomnia | 0 (0) | 2 (5.3) | 0 (0) |
| Neurological | Libido Decreased | 1 (1.1) | 0 (0) | 0 (0) |
| Neurological | Concussion | 1 (1.1) | 0 (0) | 0 (0) |
| Neurological | Somnolence | 2 (2.2) | 0 (0) | 0 (0) |
| Other | Fat Biopsy Related Complication | 2 (2.2) | 0 (0) | 0 (0) |
| Renal, urine | Proteinuria | 1 (1.1) | 0 (0) | 0 (0) |
| Renal, urine | Discoloration of Urine | 0 (0) | 1 (2.6) | 0 (0) |
| Skin | Pruritus | 2 (2.2) | 0 (0) | 0 (0) |
| Skin | Maculopapular Rash | 1 (1.1) | 1 (2.6) | 0 (0) |
| Vascular | Allergic reaction | 0 (0) | 1 (2.6) | 0 (0) |
| Vascular | Hot flash | 1 (1.1) | 1 (2.6) | 0 (0) |
| Vascular | Vascular Disorders - Hypotension | 1 (1.1) | 0 (0) | 0 (0) |
| **Total events** |  | **91** | **38** | **13** |

Data are expressed as n(%).

**Table S6. Number of serious adverse events**

|  | **Liraglutide, n** | **Placebo, n** | **Washout, n** |
| --- | --- | --- | --- |
| Chest Pain | 1 | 0 | 1 |
| Dyspnea | 0 | 1 | 0 |
| Myocardial Infarction | 2 | 0 | 0 |
| Dehydration | 0 | 1 | 0 |
| Stomach Pain | 0 | 1 | 0 |
| Skin Infection | 0 | 0 | 1 |
| Allergic reaction | 0 | 1 | 0 |
| **Total serious adverse events** | **3** | **4** | **2** |
